# Effects of Short Transport and Prolonged Fasting in Beef Calves

**DOI:** 10.3390/ani8100170

**Published:** 2018-10-03

**Authors:** Viviana Bravo, Carmen Gallo, Gerardo Acosta-Jamett

**Affiliations:** 1 Programa Doctorado en Ciencias Veterinarias, Escuela de Graduados, Facultad de Ciencias Veterinarias, Universidad Austral de Chile, Casilla 567, Valdivia, Chile; 2 Instituto de Ciencia Animal, Facultad de Ciencias Veterinarias, Universidad Austral de Chile, Casilla 567, Valdivia, Chile; cgallo@uach.cl; 3 Instituto de Medicina Preventiva Veterinaria, Facultad de Ciencias Veterinarias, Universidad Austral de Chile, Casilla 567, Valdivia, Chile; gerardo.acosta@uach.cl

**Keywords:** beef calves, transport, fasting, marketing

## Abstract

**Simple Summary:**

Marketing is inherently stressful for animals because they are removed from their home environment, handled, and transported. When sold at a livestock market, the events associated with transport are duplicated, in that animals are delivered to and then transported from the market, animals are kept confined in an unknown environment and are often mixed with unfamiliar animals, and fasting times increase. For calves, the stress of weaning is added, because the weaning process often takes place moments before being loaded for transport. In Chile, approximately one million cattle go through livestock markets annually and over 30% of them, being the largest category, are calves. Some studies have shown that calves sold through markets suffer from extended fasting periods, even when exposed to only short transportation times. The aim of this study was to determine the consequences of a short transportation time followed by an extended period without food and water. This was undertaken by measuring variables related to stress in beef calves. The results obtained showed a significant physiological effect on body temperature, blood indicators and live weight (LW). Calves lost a mean of 10 kg each after 24 h of fasting. LW loss is probably the most significant economic effect, since animals are traded based on weight. Further studies to measure the impact during true, commercial marketing are needed.

**Abstract:**

Marketing is a stressful process for beef calves, because they are removed from their environment, often weaned just before loading, loaded, transported, and unloaded. It also involves extended periods without food and water and mixing with unfamiliar animals in an unknown environment. Some studies have shown that calves sold through markets are exposed to extended fasting periods even when they undergo only short transportation times. The aim of this controlled study was to determine the consequences for beef calves of a short transportation time followed by a prolonged time without food and water on their tympanic temperature (TT), maximum eye temperature (MET), blood variables related to stress, and live weight. Ten calves were transported for 3 h and then kept in an outdoor pen for 21 h, completing a 24 h fasting period. Sampling took place before loading, after transport and unloading, and then after completing 24 h without food and water. TT, MET, blood glucose, and creatine kinase (CK) increased significantly after transportation. Live weight decreased across sample times (mean of 10 kg per calf after 24 h of fasting), which was consistent with the higher concentration of β-HB found after fasting. Further studies to measure the actual consequences of true, commercial marketing on calf welfare and productivity are needed.

## 1. Introduction

Selling cattle through livestock markets is still common in many South American countries [1,2]. At livestock markets, animals are handled by anonymous, and generally untrained, handlers [3], and are exposed to at least twice as many physical and psychological stressors than calves sold directly from farm to farm [4]. In Chile, approximately one million cattle go through livestock markets annually and over 30% of them, being the main category sold, are calves. Preliminary results [5] show that the mean transport time of calves from origin (farm) to the market is only 75 min (although it ranged between 5 min and 13 h) and from the market to the final destination (usually another farm where calves are fattened) is only 45 min (5 min–40.5 h). However, including the time spent in the holding pens (without any food or water), calves generally underwent at least 12 h of fasting, and frequently up to 24 h of fasting, which is the maximum time allowed by Chilean legislation [6,7].

Although in many countries there are now regulations regarding the transport and slaughter of livestock [1], fasting times remain long in many South American countries for diverse reasons [2]. The short- and long-term effects of transport and fasting on welfare and production indicators in calves have not attracted as much attention as they have in slaughter weight cattle, and they are also more difficult to measure. Werner et al. [8] described body weight changes and some blood constituent changes related to the stress response during a 63-h transport of recently weaned calves (approximately 240 kg) in the Chilean Patagonia. The high cortisol concentration values before transport, found by Werner et al. [8], suggest that the handling processes before transport (herding from distant fields and regrouping in pens before loading and weaning), which are common practice in extensive systems in the Patagonia, were already stressful for the calves, and represented the highest mean value found throughout all sampling stages. In the same study [8], there was significant body weight loss in the calves after the 63-h period (14% of LW, live weight). A recovery period of 30 days was required, probably because the calves ate and drank less than usual in the new environment but continued to mobilize body reserves. These results show that the long-distance transport of calves not only has an effect on animal welfare, but also creates economic losses for producers.

The marketing process is inherently stressful for calves because they are taken away from their environment, often weaned just before loading, loaded, unloaded, and transported. When selling through a livestock market additional stress can be caused because: the events associated to transport happen more than once, as they go to and from the market; animals are kept confined in an unknown environment and are often mixed with unfamiliar animals; and time without food and water increases. Physiological indicators commonly used to measure stress in animals have been reviewed by Knowles et al. [9]. Blood variables as indicators of stress have some limitations given that the handling and restraining of animals is required during sampling, which in turn produces a stress response [10].

When an animal becomes stressed, the hypothalamic–pituitary–adrenocortical (HPA) axis is activated because of the increase in catecholamines and cortisol concentrations, in addition to blood flow responses, and will produce changes in heat production and heat loss from the animal [11]. During a physical attack, or in response to a painful stimulus, blood can be diverted from the cutaneous bed and redirected to bodily organs with more urgent metabolic requirements [12]. The effect of this vasoconstriction is a decrease in skin temperature, which can be detected by infrared thermography (IRT). IRT measures the superficial temperature of objects in a non-invasive manner and has been widely used to measure the superficial body temperature in animals [13,14,15,16]. Eye temperature has been shown to be a more consistent measure of temperature change than other anatomical areas, particularly in response to stress, and is not interfered with by fur or hair [17,18,19,20,21,22,23].

This is a preliminary study with the aim of determining the consequences for beef calves of a short transportation time followed by a prolonged time without food and water on their tympanic temperature (TT), maximum eye temperature (MET) measured using IRT, blood variables related to stress, and live weight.

## 2. Materials and Methods

The Bioethics Committee “Use of animals in research” of the Universidad Austral de Chile, approved the present study (Application N°325/2018).

Ten black and red Angus calves, mixed male and female, with a mean live weight of 146.1 ± 19.1 kg, weaned a month before the experiment, and clinically healthy were used. All calves had been bred on the same farm where the experiment was performed; they were kept on pasture during the day, with water *ad libitum*, and were put in a barn overnight, receiving 1 kg/head of sugar beet pulp and hay.

The study was carried out in Lanco, Chile, during winter. The study started at 9 a.m. and the calves were calmly moved approximately 50 m from the barn to a pen, and then to a race and chute that had a head-holder for immobilization, blood sampling, and measurement of other variables. Sampling took place before loading, after transport and unloading, and again after 24 h without food and water in an outdoor pen, to simulate the conditions during commercial movement of calves for livestock markets. The transport journey had a duration of 3 h with a space allowance of 1 m^2^ per 270 kg and started approximately 2 h after the first sampling. Table 1 summarizes the environmental data present at the time the study was carried out [24].

### 2.1. Tympanic Temperature (TT)

TT was used as an indicator of body temperature. Data logger devices (ibuttons, Maxim Integrated Products Inc., San Jose, CA, USA) were manually installed into the tympanic canal using the same procedure described by Arias et al. [25]. Devices were fitted 24 h before initiating the experiment. Data loggers registered TT every 10 min; thus, in order to obtain values for the sample times (before loading, after unloading), the measurements from the 3 h before and after transport were averaged for each calf. The data from two calves and all TT records after the 24 h fasting period were omitted from the analysis as anomalous values were obtained, probably because of device displacement inside the calves’ ears.

### 2.2. Maximum Eye Temperature (MET)

**Figure 1:**
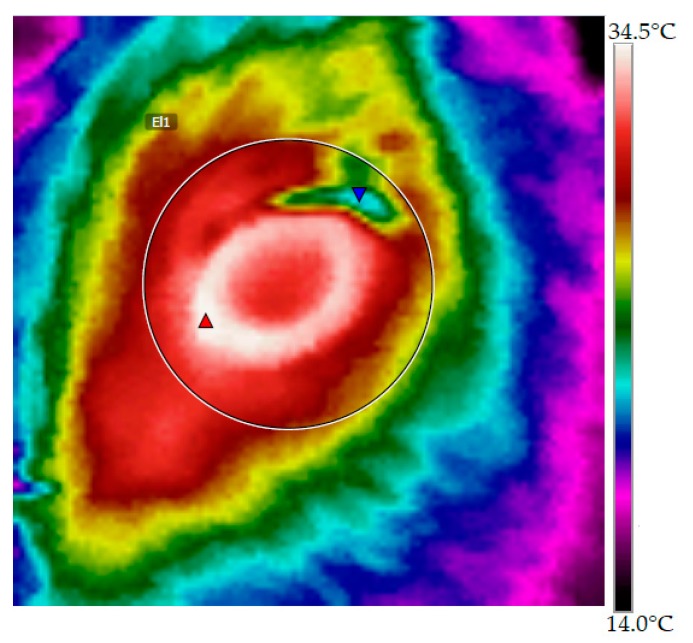
Infrared image of the eye region of a calf. Red triangle indicates maximum eye temperature (MET).

MET was obtained by capturing infrared images of both eyes at approximately 0.3 m distance and a 90° angle from the individual using a thermal camera (FLIR i5, FLIR Systems, Wilsonville, OR, USA), calibrated with an emissivity(ε) of 0.95, according to information provided by the manufacturer. Image analysis was performed using the software FLIR Tools 3.1 (FLIR Systems, Wilsonville, OR, USA) and atmospheric temperature and relative humidity were included in the calculation. The image obtained was used for analysis, as shown in Figure 1. The MET of both eyes was averaged to obtain one MET value for each calf at each sampling.

### 2.3. Blood Variables

Blood samples were collected by jugular venopunction using vacutainer^®^ (needle 20G x1"). Three collection tubes were used: A tube without additives was used for measurements of cortisol and haptoblogin (HPT) serum levels, a tube with EDTA was used for creatine kinase (CK) and betahydroxybutyrate (β-HB) measurements, and a third collection tube with NaF was used for glycemia measurements. All blood samples were placed immediately on ice, then centrifuged (2500 rpm for 5 min) for plasma or serum harvest, and stored at −20 °C for subsequent lab analysis. Plasma cortisol concentrations were determined by chemiluminescent immunoassay radioimmunoassay (CLIA) and glucose plasma concentrations were determined using the GOD-PAP test. The concentrations of β-HB were determined using the enzymatic technique that used the 3-hydroxybutyrate dehydrogenase enzyme, and plasma CK activity was measured using the IFCC and ECCLS kinetic method. Serum haptoglobin concentrations were determined using the commercial kit Tridelta PHASE Haptoglobin Assay (Tridelta Development Ltd., County Kildare, Ireland).

### 2.4. Live Weight (LW)

All calves were individually weighed after all other measurements were taken, using a mechanical cattle scale (0.5–1000 kg).

### 2.5. Data Analysis

For descriptive analyses, variables are described by mean and standard error (SE). To assess the variation in the physiological variables measured in calves, after transport and fasting times, linear mixed model (LMM) analyses were performed. The model used was:

 γij=μ+βi+εij 

where γ corresponds to a dependent variable measured; μ is time (three evaluations for each calf), considered as fixed effect; β represents the calf, included as random effect; and ε is the error not explained by the model. Data were analyzed using the lme4 statistical package and multiple comparisons were explored using a Tukey’s adjustment included in the lsmeans function in R software version 3.2.2 [26].

## 3. Results and Discussion

A significant increase of 0.55 °C in TT was found between the temperatures before loading and after unloading (Table 2). However, a 1.1 °C daily variation can occur even under moderate thermal conditions in beef cattle [27]. The body temperature represents an integrated response to both internal and external factors such as climatic conditions, heat production, and the heat losses that an animal experiences [25]. Moreover, core body temperature can also be dramatically increased with muscular activity, and by nervous and hormonal factors (such as sympathetic nervous activity), catecholamines, and thyroid hormones [28]. The relationship between body temperature and the level of stress, either physical and/or psychological, at several stages of animal handling has also been shown [29,30].

MET also increased significantly after transport and then returned to initial values after the 24 h fasting period. The rise in temperature after transport was 1.6 °C (Table 2). An increase in MET has previously been reported following castration in calves [33], after jumping competitions in horses [21], and during the veterinary clinic examination phase, compared with both pre- and post-examination phases, in dogs [23]. Nevertheless, some authors described a rapid drop in eye temperature during the minutes following a stressful and/or painful procedure, for example, calves disbudded without local anesthetic [18] and heifers exposed to fear related handling procedures such as being hit with a plastic tube, sudden flag movements, shouting, and being shocked with an electric prod [19]. The rapid drop in eye temperature in cattle during the first seconds after the stimuli has been explained as a sympathetic response, which is part of the autonomic nervous system reaction [17,18]. If the stressor persists for a longer time, the HPA axis produces a cortisol response that could be maintained from minutes to hours. In addition, the nature of the stimulus, or the level of fear and/or pain, that the animals experience may affect the duration of the drop in eye temperature [19]. The results of the present study suggest that transportation was a strong enough stimulus to cause a later increase in eye temperature in calves, as described by Stewart et al. [17,18]. However, no relationship between the increase in eye temperature and HPA axis activation has been shown [33,34]. The increases in MET and TT could reflect a body temperature increase due to stress and physical exercise during transport. It has been proposed that MET, measured with infrared thermography, may be associated with body core temperature [13,35]. In addition, although thermography is described as a non-invasive measurement, in the case of the MET measurements the animals had to be immobilized in a chute, which was undoubtedly a stressful procedure by itself. Otherwise, if there had been a drop in the eye temperature of the calves due to the handling procedures, it would probably have happened during loading, after the current measurement was made. MET in the calves decreased after unloading, returning to initial levels after the fasting period (Table 2). Cattle are a homeothermic species that need to maintain a high metabolic rate to generate heat, and so they require a high level of feed intake [36].

Regarding the blood variables; cortisol and haptoglobin did not change significantly after 3 h of transport or after 24 h of fasting (Table 2). When animals are transported, the effects of this process can be assessed by monitoring glucocorticoid concentrations, but the limitations described for this stress indicator, such as the diurnal fluctuation in plasma cortisol concentration and the effects of the sampling procedure itself, may prevent a correct evaluation [10,37]. However, all mean levels were higher than reference values for cortisol after unloading and after the 24 h fasting period, probably due to HPA axis stimulation during sampling. Animal scientists have hypothesized that glucocorticoids may have an impact on the production of acute phase proteins such as haptoglobin [9]. Literature suggests that haptoglobin is less sensitive and slower reacting than other acute phase proteins, like serum amyloid A (SAA) [38,39]. Werner et al. [8] showed a significant increase in haptoglobin levels 24 h after unloading beef calves transported for a duration of 63 h.

Creatine kinase increased significantly after transport and returned to initial values during fasting in pens (Table 2). CK appears in the circulating plasma because of tissue damage (e.g., bruising), and also when there is vigorous exercise, and is relatively organ specific (i.e., muscle) [37]. In this study, calves were transported at a low stocking density which probably meant that an additional physical effort from calves was required in order to maintain balance during transport. Additionally, this same factor could have influenced bruising when the truck was in motion. The significant decrease in CK after the 24 h fasting period is likely associated with the elimination of the physical stressor, and a rise in excretion, as observed previously in steers after transport for slaughter [40]. CK values were higher than reference values (Table 2) at all sampling times, possibly because of previous handling, such as immobilization for sampling.

After strenuous exercise, ketone oxidation by muscles is reduced and this leads to an increase in plasma levels of betahydroxibutyrate [9]. Additionally, the high weight loss observed was consistent with the significantly higher concentration of β-HB found after the 24 h fasting period (with transport included). This indicated that, even with the short time off feed, the calves had to make use of their body reserves, even though mean β-HB values were within normal ranges for the species for all sample times (Table 2).

Blood glucose levels reflect the nutritional status of an animal, and during food deprivation the glucose blood levels decrease [41]. The results here show that transport may have had a greater impact on plasma glucose than food deprivation (Table 2). There were significant differences in glucose values between sampling times (Table 2). Glycemia increased over the reference values after transport and then returned to basal levels, but remained higher than before loading. The maintenance of glycemia may have been associated with the use of liver glycogen, as practically all glucose available to ruminants comes from the glycogenolysis process [42,43]. Increases in glucose levels after transportation and fasting have been previously reported in calves after 8 h of fasting and 8 h of transport [44]. The explanation for this increase is related to the primary response to stress [9], so glucose levels can be used as an indirect indicator of stress [41].

The calves lost 10 kg live weight (LW). Fifty percent of the total LW loss occurred between sampling before loading and sampling after unloading (within approximately 5 h), whereas the other 50% occurred while the calves were fasting in the pen (Table 2). Generally, weight loss during transport is accelerated compared with deprivation of food and water without transport [9]. The calves lost 6.8% of LW in 24 h, which is consistent with Knowles et al. [9] who described an initial loss of LW in ruminants, predominantly due to loss of gut fill, of approximately 7% during the first 18 to 24 h without food and water. Metabolic changes due to stress are greatest if increased heat production and decreased feed intake occur concomitantly. In this situation, the required calories come from the catabolism of tissues [45]. Body weight loss in animals is probably the most significant economic effect of transport [8].

## 4. Conclusions

From this preliminary study it can be concluded that even in a controlled replication of the marketing process, where calves were weaned a month before the study, were handled calmly, transported at a low stocking density, and fasted without being mixed or exposed to an unfamiliar environment, there was a significant effect of transport and fasting on the calves, which were measurable through MET, TT, blood indicators such as CK, glucose, β-HB and their live weight. This study was limited to a small number of animals, lacked a control group, and was under simulated commercial conditions. Nevertheless, the findings strongly suggest that more extensive studies of calves undergoing true, commercial marketing are urgently required. Exposure to a real marketing situation, with additional real-life stressors such as handling by untrained staff, weaning just before loading or when they arrive in the market, transport both to and from the market, fasting in pens without protection against adverse weather conditions, stocking at higher densities, and getting mixed with unfamiliar animals would undoubtedly produce greater stress, with repercussions for calf welfare and productivity.

## Figures and Tables

**Table 1:** Values for climatic variables during the study period.

Time	AT Min (RH)–Max (RH) ^1^
Before loading	3.5 (94.8)–7.3 (81.6)
During transport	7.3 (81.6)–11.6 (67)
After unloading	10.3 (80.2)–11.4 (71.5)
During fasting	6.9 (89.8)–10.3 (80.2)
After 24 h fasting	8 (94.8)–8.3 (92.6)

^1^ AT: Air temperature (°C); RH: Relative humidity (%).

**Table 2:** Mean (±SE) of all the variables measured before loading, after unloading, and after the 24-h fasting period.

Variable	Before Loading	After Unloading	After Fasting	Reference *
Live weight (kg)	146.1 (±6.0) ^a^	141.1 (±5.9) ^b^	136.1 (±5.9) ^c^	-
Tympanic T° (°C)	37.15 (±0.21) ^a^	37.7 (±0.10) ^b^	-	-
Maximum eye T° (°C)	34.72 (±0.22) ^a^	36.30 (±0.17) ^b^	34.48 (±0.19) ^a^	-
Cortisol (µg/dL)	1.55 (±0.34) ^a^	2.28 (±0.35) ^a^	2.29 (±0.36) ^a^	0.3–2.0
Glucose (mmol/L)	3.68 (±0.10) ^a^	4.38 (±0.14) ^b^	3.97 (±0.10) ^c^	2.5–4.1
β-HB (mmol/L)	0.26 (±0.02) ^a^	0.22 (±0.03) ^a^	0.35 (±0.04) ^b^	0.1–0.6
Creatine kinase (U/L)	242.9 (±18.9) ^a^	398.4 (±46.6) ^b^	189.3 (±15.5) ^a^	<94
Haptoglobin (mg/mL)	0.16 (±0.01) ^a^	0.15 (±0.00) ^a^	0.26 (±0.07) ^a^	<3 mg/mL

Different letters (a, b, c) represent statistical differences (*p* < 0.05) between sampling times. * Reference values: for cortisol according to DCPAH, Michigan State University [31]; other blood variables according to Wittwer [32].
